# High-Throughput Transcriptomics Identifies Chemoresistance-Associated Gene Expression Signatures in Human Angiosarcoma

**DOI:** 10.3390/ijms251910863

**Published:** 2024-10-09

**Authors:** Glenys Mai Shia Khor, Sara Haghani, Tiffany Rui En Tan, Elizabeth Chun Yong Lee, Bavani Kannan, Boon Yee Lim, Jing Yi Lee, Zexi Guo, Tun Kiat Ko, Jason Yongsheng Chan

**Affiliations:** 1 Cancer Discovery Hub, National Cancer Centre Singapore, 30 Hospital Blvd, Singapore 168583, Singapore; expoexperiment@yahoo.com (G.M.S.K.);; 2 Raffles Institution, 1 Raffles Institution Ln, Singapore 575954, Singapore; 3 Duke-NUS Medical School, 8 College Rd, Singapore 169857, Singapore; 4 Division of Medical Oncology, National Cancer Centre Singapore, 30 Hospital Blvd, Singapore 168583, Singapore

**Keywords:** angiosarcoma, whole transcriptome sequencing, chemoresistance, secreted phosphoprotein 1, immune-oncology

## Abstract

Angiosarcomas, clinically aggressive cancers of endothelial origin, are a rare subtype of soft-tissue sarcomas characterized by resistance to chemotherapy and dismal prognosis. In this study, we aim to identify the transcriptomic biomarkers of chemoresistance in angiosarcoma. We examined 72 cases of Asian angiosarcomas, including 35 cases treated with palliative chemotherapy, integrating information from NanoString gene expression profiling, whole transcriptome profiling (RNA-seq), immunohistochemistry, cell line assays, and clinicopathological data. In the chemoresistant cohort (defined as stable disease or progression), we observed the significant overexpression of genes, including *SPP1* (log2foldchange 3.49, adj. *p* = 0.0112), *CXCL13*, *CD48*, and *CLEC5A*, accompanied by the significant enrichment of myeloid compartment and cytokine and chemokine signaling pathways, as well as neutrophils and macrophages. RNA-seq data revealed higher *SPP1* expression (*p* = 0.0008) in tumor tissues over adjacent normal compartments. Immunohistochemistry showed a significant moderate positive correlation between *SPP1* protein and gene expression (r = 0.7016; *p* < 0.00110), while higher *SPP1* protein expression correlated with lower chemotherapeutic sensitivity in patient-derived angiosarcoma cell lines MOLAS and ISOHAS. In addition, *SPP1* mRNA overexpression positively correlated with epithelioid histology (*p* = 0.007), higher tumor grade (*p* = 0.0023), non-head and neck location (*p* = 0.0576), and poorer overall survival outcomes (HR 1.84, 95% CI 1.07–3.18, *p* = 0.0288). There was no association with tumor mutational burden, tumor inflammation signature, the presence of human herpesvirus-7, ultraviolet exposure signature, and metastatic state at diagnosis. In conclusion, *SPP1* overexpression may be a biomarker of chemoresistance and poor prognosis in angiosarcoma. Further investigation is needed to uncover the precise roles and underlying mechanisms of *SPP1*.

## 1. Introduction

Angiosarcoma (AS), a clinically aggressive cancer of endothelial origin, is a rare subtype of soft-tissue sarcoma with dismal prognosis [1,2]. AS tumors exhibit significant clinical and genetic heterogeneity and can originate in various anatomical sites, most commonly in the head and neck, breasts, and extremities [3]. The treatment of AS is notoriously challenging, with low survival rates despite multimodal approaches combining radical surgical resection with radiotherapy and chemotherapy [2,4]. In particular, antineoplastic agents, such as paclitaxel and doxorubicin, are often hindered by primary resistance or produce only transient responses, followed by the rapid emergence of acquired drug resistance [5]. Excluding clinical factors such as patient age, disease stage, and histopathological characteristics [6,7,8], there remains a lack of reliable biomarkers to predict survival outcomes and chemotherapy responsiveness, which would significantly enhance patient selection for targeted treatment.

At the molecular level, gene expression profiling has revealed three distinct AS clusters represented by the lack or enrichment of immune-related signaling and immune cells, as well as varying tumor mutation burden and tumor inflammation signature scores [9]. In addition, spatial transcriptomics has revealed topological profiles of the tumor microenvironment [9], while extensive genomic profiling has uncovered multiple actionable mutations, underscoring the promise of precision medicine in AS treatment [10]. However, while multi-omic analyses and immune profiling studies have revealed the genomic and topological immune landscapes of angiosarcoma, the molecular mechanism of chemoresistance in AS has yet to be elucidated.

Secreted phosphoprotein 1 (*SPP1*), also known as osteopontin (OPN), is a small integrin-binding ligand, N-linked glycoprotein (SIBLING) located at 4q22.1. This highly acidic secreted phosphoprotein has a diverse range of functions [11], including bone regeneration [12], angiogenesis [13], cell adhesion and migration [14], and inflammation [15]. Emerging evidence has shown that the high *SPP1* expression is associated with poor prognosis in multiple cancer types [16]. Many studies have described the overexpression of *SPP1* in multiple cancers such as breast carcinoma [17], hepatocellular carcinoma [18], penile cancer [19], ovarian carcinoma [20], and lung adenocarcinoma [21], as well as the key roles of *SPP1* in invasion, metastasis, chemoresistance, and immune suppression [22,23]. In lung adenocarcinoma, the upregulation of PD-L1 by *SPP1* has been shown to mediate macrophage polarization, facilitate immune escape [24], and act as an immune checkpoint that induces host tumor immune tolerance by suppressing T cell activation [25]. However, the role of *SPP1* in AS remains unclear. Hence, transcriptome analysis may elucidate molecular mechanisms underlying chemoresistance, as well as the role of *SPP1* in angiosarcoma, aiding in the identification of novel biomarkers for targeted therapies for angiosarcoma.

In this study, we examined *SPP1* expression in angiosarcoma and its role in chemoresistance using NanoString gene expression data, immunohistochemistry, and in vitro testing. Additionally, we seek to investigate potential correlations among *SPP1* expression, clinicopathological features, immuno-oncologic pathways, and patient survival outcomes.

## 2. Results

### 2.1. Patient Cohort

Our study included a total of 72 patients, consisting of 47 men (65.3%) and 25 women (34.7%), with a median age of 65.7 years (range, 27.8–92.9 years). The majority of these patients were of Chinese ethnicity (80.6%). The majority of cases (n = 42, 58.3%) were diagnosed as primary angiosarcoma originating from the head and neck. Additionally, 51 cases (70.8%) exhibited signatures of UV DNA damage, and 25 cases (34.7%) tested positive for human herpesvirus-7. At the time of diagnosis, 22 cases (31.0%) were identified as metastatic. A summary of patient characteristics can be found in Table 1.

### 2.2. NanoString Gene Expression Profiling

**Figure 1:**
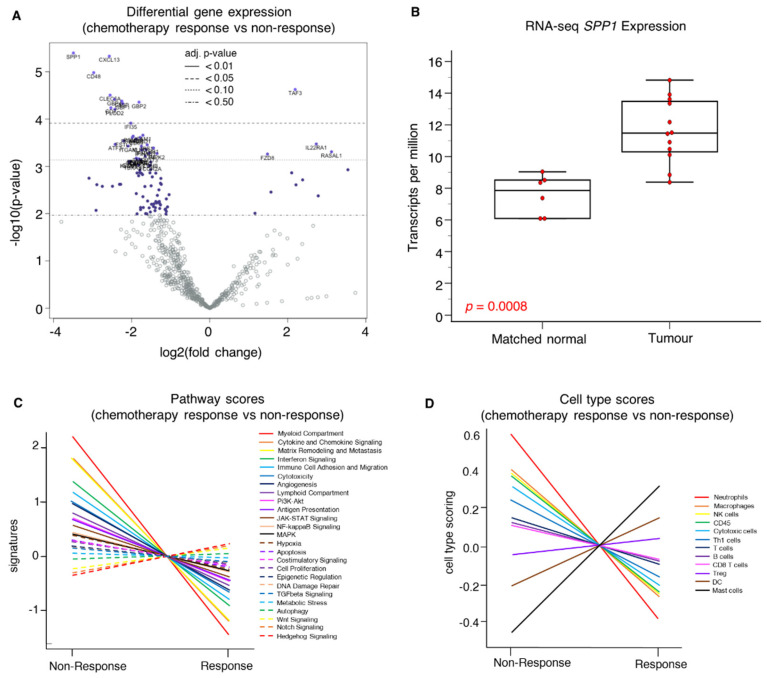
Transcriptomic analysis of chemoresistant versus chemosensitive angiosarcoma. (**A**) Volcano plot of the differential gene expression in chemoresistant versus chemosensitive angiosarcoma reveals significant *SPP1* overexpression in chemoresistant angiosarcoma. (**B**) Significant upregulation of *SPP1* expression in tumor compared to matched normal tissue reflected in whole transcriptome sequencing data. (**C**) Pathway analysis showed that expression of myeloid compartment, cytokine and chemokine signaling, and matrix remodeling and metastasis pathways were upregulated in chemoresistant tumors. Conversely, Hedgehog, Notch, and Wnt signaling pathways were upregulated in chemosensitive tumors. (**D**) Cell-type analysis of chemoresistant tumors suggested enrichment of myeloid cells including neutrophils, macrophages, and NK cells, whereas a greater population of mast cells, dendritic cells (DC), and regulatory T cells (Treg) were observed in chemosensitive tumors.

The top four genes that were significantly overexpressed in non-responders to palliative chemotherapy were *SPP1* (log2foldchange 3.49, adj. *p* = 0.0112), *CXCL13* (log2foldchange 2.57, adj. *p* = 0.0112), *CD48* (log2foldchange 2.97, adj. *p* = 0.0166), and *CLEC5A* (log2foldchange 2.55, adj. *p* = 0.0247). Conversely, *TAF3* was significantly overexpressed (adj. *p* < 0.05) in chemosensitive tumors (Figure 1A and Supplementary Table S1). To survey the transcriptomic landscape further on a global level, we evaluated available whole transcriptome RNA-seq data of a subset of samples in tumor (n = 12) and matched normal tissue (n = 6) from our previous study [2]. The expression of the top candidate biomarker, *SPP1*, was significantly higher in tumor tissue compared to matched normal tissue (*p* = 0.0008) (Figure 1B) in keeping with NanoString gene expression analysis.

**Figure 2:**
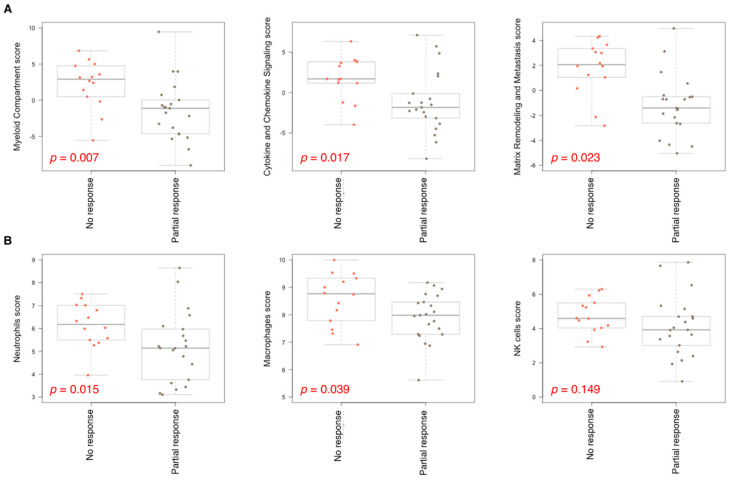
Chemotherapy response of angiosarcoma correlates with immuno-oncogenic pathways and cell types. (**A**) Significance of association of top three immuno-oncology pathways with chemotherapy non-response versus response in angiosarcoma. (**B**) Significance of association of top three cell types with chemotherapy non-response versus response in angiosarcoma.

A pathway analysis indicated the upregulation of the myeloid compartment (*p* = 0.007), cytokine and chemokine signaling (*p* = 0.017), and metastasis/matrix remodeling pathways in chemoresistant tumors. Conversely, Hedgehog, Notch, and Wnt signaling pathways were upregulated in chemosensitive tumors (Figure 1C). A cell-type analysis suggested that myeloid cells, including neutrophils (*p* = 0.015) and macrophages (*p* = 0.039), along with NK cells (*p* = 0.149), were enriched in chemoresistant tumors (Figure 1D), whereas a greater population of mast cells, dendritic cells (DC), and regulatory T cells (Treg) were observed in chemosensitive tumors (Figure 1D). Figure 2 illustrates selected pathways and cell types.

**Figure 3:**
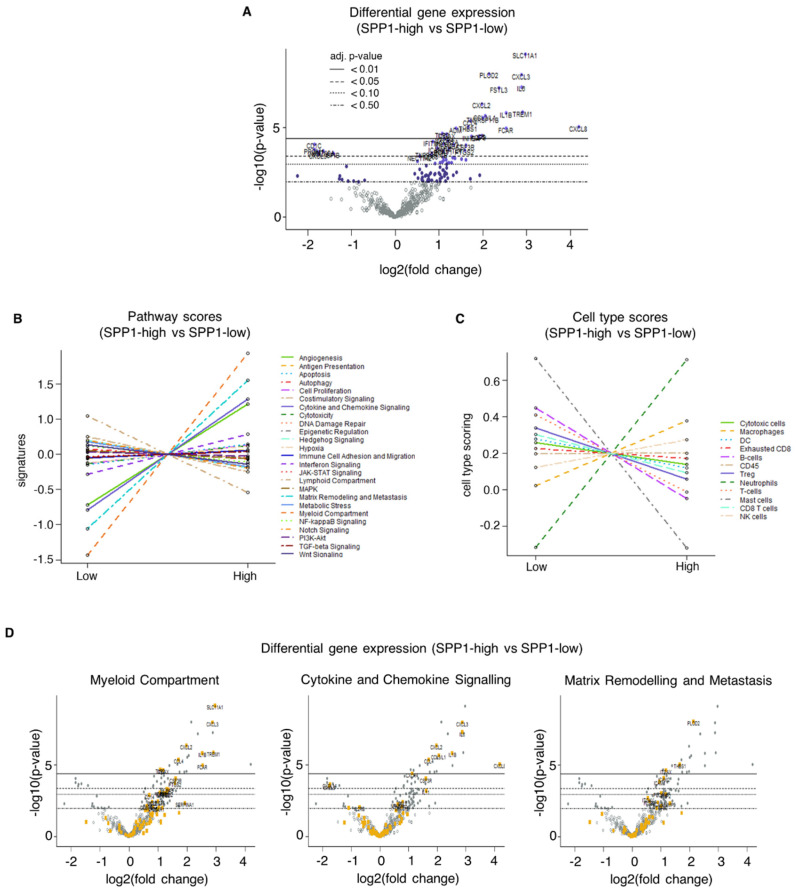
Transcriptomic analysis of *SPP1*-high and *SPP1*-low angiosarcoma. (**A**) Volcano plot of differentially expressed genes between *SPP1*-high and *SPP1*-low tumors (n = 72) revealed significant overexpression of various genes, including *SLC11A1*, *PLOD2*, *CXCL3*, *IL6*, *FSTL3*, *CXCL2*, *TREM1* and *IL1β*. (**B**–**D**) NanoString pathway analysis and gene-specific analysis (GSA) revealed significant overexpression of myeloid compartment pathway genes (*SLC11A1*, *CXCL3*, *CXCL2*, *TREM1*, *IL1β*), cytokine and chemokine signaling pathway genes (*CXCL3*, *IL6*, *CXCL2*, *IL1β*), and matrix remodeling and metastasis pathway genes (*PLOD2*). (**C**) Cell-type analysis of *SPP1*-high tumors suggested enrichment of myeloid cells, including neutrophils, macrophages, and NK cells.

Upon further evaluation of all 72 samples, we observed differentially expressed genes between *SPP1*-high and *SPP1*-low tumors (n = 41 for *p* < 0.05, n = 23 for *p* < 0.01) (*p*-values adjusted for false discovery rate following Benjamini–Yekutieli procedure). Genes that were significantly overexpressed (*p* < 0.001) included *SLC11A1*, *PLOD2*, *CXCL3*, *IL6*, *FSTL3*, *CXCL2*, *TREM1*, and *IL1β* (Figure 3A and Supplementary Table S2). A gene-specific analysis (GSA) revealed the significant overexpression (*p* < 0.001) of myeloid compartment pathway genes (*SLC11A1*, *CXCL3*, *CXCL2*, *TREM1*, *IL1β*), cytokine and chemokine signaling pathway genes (*CXCL3*, *IL6*, *CXCL2*, *IL1β*), and matrix remodeling and metastasis pathway genes (*PLOD2*). A cell-type analysis of *SPP1*-high tumors was suggestive of the enrichment of neutrophils, macrophages, and NK cells, similar to that in chemoresistant tumors (Figure 3B–D).

#### 2.2.1. Association of SPP1 mRNA Expression with Clinical Parameters

**Figure 4:**
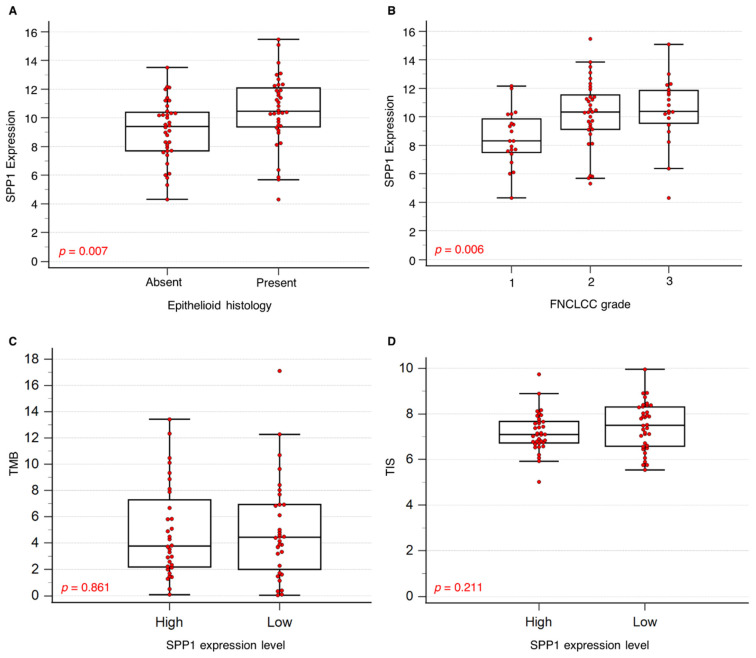
Clinicopathological characteristics associated with *SPP1* expression in angiosarcoma. (**A**) Presence of epithelioid histology was significantly associated with higher *SPP1* gene expression levels. (**B**) Higher FNCLCC grading was significantly associated with higher *SPP1* gene expression levels. (**C**,**D**) Tumor mutational burden (TMB) and tumor inflammatory signature (TIS) scores were not associated with *SPP1* gene expression levels.

*SPP1* mRNA overexpression emerged as a significant candidate biomarker of chemoresistance and was positively correlated to the presence of epithelioid features (*p* = 0.007) and high FNCLCC histological tumor grade (*p* = 0.006) (Figure 4A,B). Dichotomizing into high or low scores using the median *SPP1* expression value of 10.23, high *SPP1* expression was correlated with non-head and neck location (*p* = 0.0576), but no significant association was found with TMB, TIS scores, the presence of human herpesvirus-7, ultraviolet exposure signature, or metastatic state at diagnosis (Figure 4C,D) (Table 1).

#### 2.2.2. Spatial Analysis of Breast and Scalp Angiosarcoma

**Figure 5:**
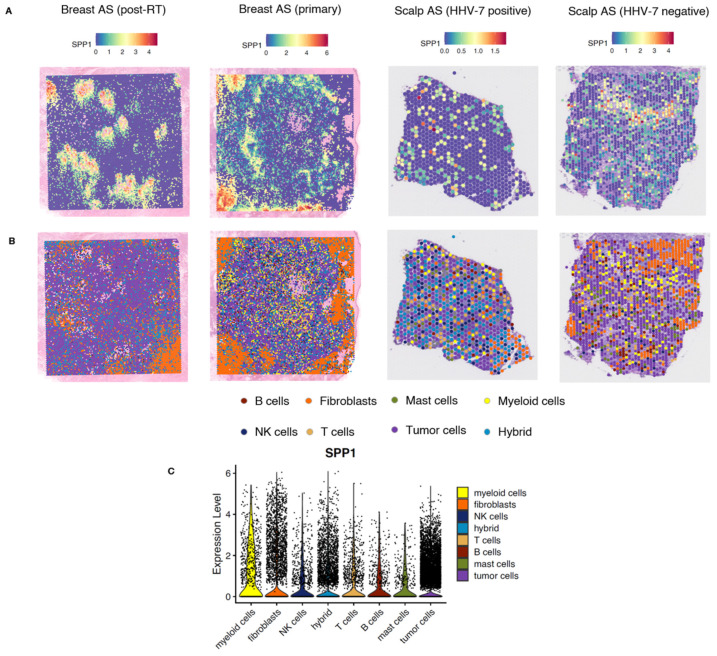
Spatial distribution of *SPP1*. (**A**) Spatial transcriptomics analysis showed that *SPP1* expression displayed significant heterogeneity in terms of spatial distribution in all samples, with focal clusters of cells expressing higher levels of *SPP1* scattered throughout the tumor tissues. (**B**) Spatial distribution of various cell types in the tumor tissues. (**C**) Violin plots showing *SPP1* expression across all cell types in angiosarcoma, including stromal, immune, and tumor cells, and it was most enriched in myeloid cells.

Subsequently, we used 10× Genomics Visium spatial transcriptomics to analyze four samples (two angiosarcomas of the breast and two of the head and neck). A total of 28,988 55-micron spots were analyzed (primary breast AS, 13101; post-RT breast AS, 13600; HHV7-positive scalp AS, 748; HHV7-negative scalp AS, 1539). A spatial transcriptomics analysis showed that *SPP1* expression displayed significant heterogeneity in terms of spatial distribution in all samples, with focal clusters of cells expressing higher levels of *SPP1* scattered throughout the tumor tissues (Figure 5A). *SPP1* expression was present across all cell types in angiosarcoma, including stromal, immune, and tumor cells, and in corroboration with NanoString immune profiling, was most enriched in myeloid cells (Figure 5).

#### 2.2.3. Immunohistochemical Staining for SPP1 Protein Expression

**Figure 6:**
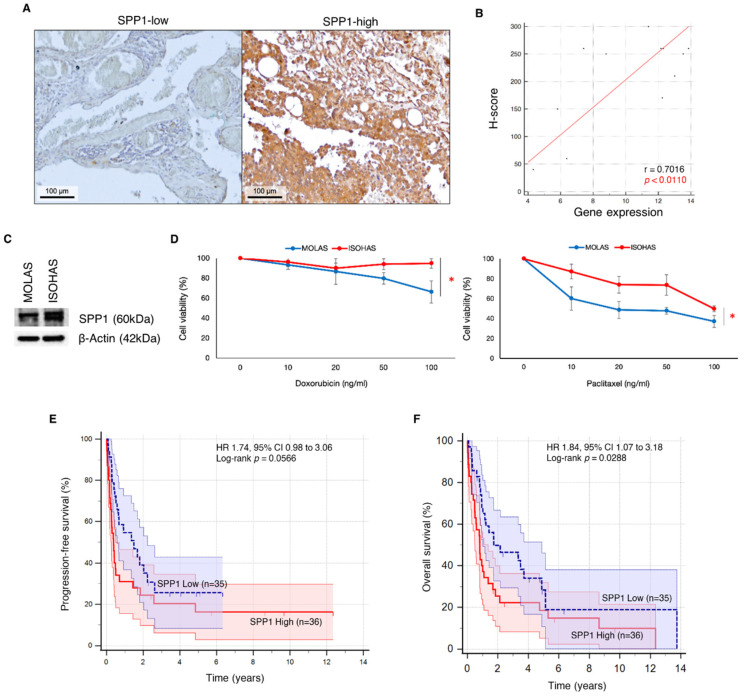
Prognostic implications of *SPP1*. (**A**) IHC images at 400× magnification (scale bar, 100 µM) of tissue sections stained with anti-osteopontin antibody (*SPP1*). Representative images for high and low *SPP1* H-scores are shown. (**B**) Scatterplot showing significant moderate correlation between *SPP1* expression levels from IHC H-scores and NanoString (Spearman’s r = 0.7016; *p* < 0.0110). (**C**) Western blot reflected higher *SPP1* protein expression in ISOHAS as compared to MOLAS cell line. (**D**) In the patient-derived MOLAS and ISOHAS cell lines, treatment with paclitaxel and doxorubicin resulted in reduced viability in a dose-dependent manner, with ISOHAS demonstrating higher cell viability than MOLAS upon treatment with either chemotherapeutic drug. *, *p* < 0.05. (**E**,**F**) Kaplan–Meier curves showing survival probability in patients with *SPP1*-high versus *SPP1*-low angiosarcoma. Patients with *SPP1*-high angiosarcoma showed poorer overall survival (CI 1.07 to 3.18, HR 1.84, *p* = 0.0288), along with a trend toward poorer progression-free survival (CI 0.98 to 3.06, HR 1.74, *p* = 0.0566) compared to patients with *SPP1*-low angiosarcoma.

We examined *SPP1* expression in the angiosarcoma tumor archival FFPE samples using immunohistochemistry (Figure 6A). A significant moderate positive correlation (r = 0.7016; *p* < 0.0110) was observed between *SPP1* expression levels obtained via immunohistochemistry and NanoString gene profiling (Figure 6B).

#### 2.2.4. In Vitro Response to Chemotherapeutic Drugs

Western blot analyses demonstrated higher protein expression of *SPP1* in ISOHAS compared to MOLAS (Figure 6C). MOLAS and ISOHAS cell lines were exposed to increasing concentrations of paclitaxel and doxorubicin (10, 20, 50, and 100 ng/mL for 72 h). Both drugs resulted in a dose-dependent reduction in cell viability in the angiosarcoma cell lines, although ISOHAS displayed greater chemoresistance compared with MOLAS (Figure 6D).

In keeping with the abovementioned data, *SPP1* gene expression was correlated with poorer overall survival (HR 1.84, 95% CI 1.07–3.18, *p* = 0.0288) and poorer progression-free survival (HR 1.74, 95% CI 0.98–3.06, *p* = 0.0566) (Figure 6E,F).

## 3. Discussion

This study demonstrated that *SPP1* overexpression is significantly associated with chemoresistance and poorer survival outcomes in human angiosarcoma (AS). Next-generation molecular diagnostics have only recently begun to characterize the pathobiology of angiosarcoma, which is an otherwise enigmatic disease with an aggressive clinical phenotype [2]. Moreover, despite recent studies into combination therapies in the management of AS, response to first-line systemic therapy is poor because of the absence of predictive biomarkers and significant intra-tumor heterogeneity [26]. Thus far, apart from one study that has employed a morphological approach to identify a potential marker of chemoresistance in AS, that is CD31^low^ cells, which relies on enhanced YAP signaling to improve redox status and is doxorubicin-resistant in AS [27], to our knowledge, this is the first study to identify the potential biomarkers of chemoresistance in AS via an omics approach.

NanoString gene expression profiling identified a gene expression signature associated with chemoresistance in AS. The most significantly upregulated gene in the gene expression signature was secreted phosphoprotein 1 (*SPP1*), which is well known to be involved in the regulation of many tumor-associated biological processes, including tumorigenesis, tumor progression, and the tumor immune microenvironment. *SPP1* has been suggested as a potential target for evaluating prognosis and immunotherapy in multiple human cancers [28]. In ovarian tumor tissues, *SPP1* overexpression was significantly associated with poor survival and indicated higher levels of immune cell infiltration [29]. In head and neck squamous cell carcinoma (HNSCC), *SPP1*^+^*CCL18*^+^ and *SPP1*^+^FOLR2^+^ tumor-associated macrophages (TAMs) harbored pro-angiogenic and metastatic transcriptional programs and were correlated with poor survival [30].

Moreover, *SPP1* overexpression has also been associated with resistance to chemoradiotherapy in multiple cancers [31]. For example, in lung adenocarcinoma, *SPP1* expression on TAMs has been found to correlate with poor prognosis and chemoresistance [32]. In ovarian cancer, the *SPP1*-CD44 axis facilitated cancer cell chemoresistance via PI3K/AKT signaling and ATP-binding cassette (ABC) drug efflux transporter activity [33]. Similarly, fibroblast-derived *SPP1* was found to contribute to resistance of hepatocellular carcinoma to sorafenib and lenvatinib treatment [34], and in oral squamous cell carcinoma, the *SPP1*-integrin αvβ3 axis was found to be crucial for 5-fluorouracil resistance [35]. Most recently, *Helicobacter pylori* infection-induced *SPP1* activation was found to promote chemoresistance and T cell inactivation in gastric cancer cells [36]. The role of *SPP1* in cancer thus appears to vary according to cancer subtype, suggesting the need for further research tailored to specific cancer types to explore its clinical implications.

In our study, we showed a correlation between *SPP1* overexpression and the upregulation of the myeloid compartment, cytokine and chemokine signaling, and metastasis/matrix remodeling pathways in chemoresistant tumors, which is in line with the cell-type analysis that suggested the enrichment of myeloid cells (neutrophils and macrophages), as well as NK cells. This finding aligns with our earlier study that demonstrated a positive correlation between the intra-tumoral neutrophil-to-lymphocyte ratio in angiosarcoma and oncogenic pathway scores, including angiogenesis, matrix remodeling, metastasis, and cytokine and chemokine signaling, along with myeloid compartment scores. These pathway scores were significantly higher in non-responders compared to responders to first-line chemotherapy [6].

Several genes in our analysis were differentially expressed between *SPP1*-high and *SPP1*-low AS tumors. A gene-specific analysis (GSA) characterized the top *SPP1*-mediated pathways in AS, including the myeloid compartment pathway with the significant overexpression of *SLC11A1*, *CXCL3*, *CXCL2*, *TREM1*, *IL1β*, and *TLR2* genes, as well as the cytokine and chemokine signaling pathway, with significant overexpression of the *CXCL3*, *IL6*, *CXCL2*, *IL1β* genes. Notably, *IL1β*-induced *IL6* and *sIL6R* have been found to trigger *IL6* trans-signaling, contributing to the upregulation of SPP1 in THP-1 macrophages [37]. Similarly, in primary lung fibroblasts, the expression of *SPP1* is potently upregulated by *IL1β* [38]. Overall, the overexpression of *SPP1* in AS could potentially be mediated by *IL1β*-induced *IL6* trans-signaling.

The limitations of our study include its retrospective approach and relatively small sample size. Additionally, tumor heterogeneity and potential sampling issues could influence the reliability of the findings, since this will affect gene expression signatures and immune profiles. Nevertheless, we have presented preliminary evidence supporting *SPP1* as an indicator of chemoresistance in angiosarcoma and investigated its potential clinical significance. The contribution of *SPP1* overexpression to chemotherapy resistance will need to be validated in a larger independent prospective study, and the exact mechanisms dissected through functional studies. In conclusion, this study illustrates *SPP1* overexpression in AS and its potential role as both a predictive biomarker of chemoresistance and a prognostic biomarker in angiosarcoma.

## 4. Materials and Methods

### 4.1. Patient Cohort

Patients with histologically confirmed angiosarcoma treated at Singapore General Hospital (SGH) and National Cancer Centre Singapore (NCCS) between January 2000 and December 2020 were selected for inclusion in our study. Certified pathologists reviewed all cases, with diagnoses corroborated by immunohistochemical staining for vascular markers such as CD31 and/or ERG. Cases of Kaposi sarcoma, epithelioid haemangioendothelioma, and intimal sarcoma were excluded. The median follow-up duration for the entire cohort was 1.0 year. Patient age, sex, and ethnicity were verified against participants’ National Registration Identity Cards. All data were collected at diagnosis or during follow-up. Clinicopathologic characteristics of the patient cohort are provided in Table 1.

#### 4.1.1. NanoString Gene Expression Profiling

The NanoString PanCancer IO360 panel (NanoString Technologies, Seattle, WA, USA) was used to perform gene expression profiling on formalin-fixed, paraffin-embedded (FFPE) tissue on the nCounter platform, following the manufacturer’s protocol. RNA extraction was conducted from five 10-µm sections of all samples containing sufficient tumor tissue, which were subsequently analyzed with the 2100 Bioanalyzer (Agilent Technologies, Palo Alto, CA, USA). From the final cohort, a subset of patients from the final cohort who were treated with palliative chemotherapy (n = 35), including paclitaxel (n = 28), liposomal doxorubicin (n = 2), doxorubicin plus ifosfamide (n = 2), doxorubicin (n = 1), ifosfamide (n = 1), and doxorubicin and cisplatin plus paclitaxel (n = 1), was analyzed on the nSolver 4.0 Advanced Analysis module using the default settings to derive differentially expressed genes, pathway scores, and cell-type scores associated with chemotherapy response to determine candidate biomarkers of chemoresistance (defined as stable disease or progression). The final cohort (n = 72) was subsequently reanalyzed to derive differentially expressed genes, pathway scores, and cell-type scores associated with the expression of the top candidate biomarker identified in the initial analysis.

#### 4.1.2. Immunohistochemistry Staining

FFPE tissue samples were procured from the Department of Pathology, Singapore General Hospital. *SPP1* staining was performed using an anti-osteopontin antibody [RM1018] (ab283656, Abcam, Cambridge, UK). The ImmPRESS Universal PLUS Polymer Kit (catalog #MP-7800; Vector Laboratories, Newark, CA, USA) was used to stain the slides according to the manufacturer’s protocol. FFPE sections underwent deparaffinization and rehydration using limonene and ethanol, and high-temperature-induced epitope retrieval was performed using a citrate-based buffer (pH 6.0) through 5 min of pressurized heating at 120 °C in a pressure cooker. The slides were incubated with BLOXALL blocking solution for 10 min to quench endogenous peroxidase and alkaline phosphatase activity, followed by treatment with 2.5% prediluted horse serum for 20 min. The slides were then incubated overnight at 4 °C with the anti-osteopontin antibody diluted to 1:200. Staining with substrate–chromogen mix was performed on the slides for 2 min, followed by counterstaining with Hematoxylin QS Counterstain (Vector Laboratories, CA, USA). *SPP1* expression was quantified by two independent readers blinded to each other and the NanoString and clinical data. Histopathologic appearances were located through images taken at 10× objective (magnification of 100×), and a more detailed evaluation was conducted at 40× objective (magnification of 400×). A histochemical score (H-score) was assigned according to the staining intensity observed in representative sections, with scores ranging from 0% to 100%.

#### 4.1.3. Western Blotting

Cells were harvested and washed with PBS. Lysis buffer was added to the cell pellet and stored at −20 °C overnight. Cell lysates were separated based on molecular weight using SDS-PAGE with 4–15% Mini-PROTEAN^TM^ TGX Stain-Free^TM^ Protein Gels (Bio-Rad Laboratories, Hercules, CA, USA). The blot was transferred onto 0.2 μm PVDF membranes (Bio-Rad Laboratories, Hercules, CA, USA) and blocked with 5% non-fat dry milk (Bio-Rad Laboratories, Hercules, CA, USA) in TBST solution (50 mM Tris/HCl pH 7.4, 150 mM NaCl, 0.1% Tween-20) for 1 h. Subsequently, the blot was left to roll at 4 °C overnight in anti-osteopontin antibody [RM1018] (ab283656, Abcam, Cambridge, UK) at 1:1000 dilution and β-Actin antibody (#4970S, Cell Signalling Technology, Danvers, MA, USA). The blot was incubated in secondary antibodies for 1 h and subsequently exposed to chemiluminescence detection using SuperSignal Substrate Western Blotting Kit (Thermo Fisher Scientific, Waltham, MA, USA). Images were taken using ChemiDocTM XRS+ System with image Lab^TM^ Software, version 5.0 (Bio-Rad Laboratories, Hercules, CA, USA).

#### 4.1.4. Cell Lines and Cell Viability Assays

Two angiosarcoma cell lines (MOLAS and ISOHAS) were obtained from the Cell Resource Center for Biomedical Research, Institute of Development, Aging and Cancer, Tohoku University, Japan, courtesy of Dr. Mikio Masuzawa. MOLAS was established from a patient with scalp lymphangiosarcoma metastatic to the pleura, while ISOHAS was established from a patient with primary scalp hemangiosarcoma. Both cell lines were maintained in a DMEM medium supplemented with 10% FBS and 1% penicillin/streptomycin. These cells were grown in a humidified chamber with 5% CO_2_ at 37 °C. To examine the response to chemotherapeutic agents, MOLAS and ISOHAS cultures were exposed to paclitaxel and doxorubicin (Selleck Chemicals, Houston, TX, USA) at concentrations of 10, 20, 50, and 100 ng/mL for 72 h. The Quick Cell Proliferation Assay Kit II (ab65475, Abcam, Cambridge, UK) was used to quantify the overall cell viability after treatment with the respective agents. Cell cultures at approximately 70% confluence were used for all experimental drug treatments unless otherwise stated.

#### 4.1.5. 10× Genomics Visium Platform

Formalin-fixed, paraffin-embedded (FFPE) tissue blocks of two breast angiosarcoma samples and two fresh frozen angiosarcoma samples of head and neck origin were examined for *SPP1* expression pattern. The AS breast samples were processed on the Visium CytAssist (10× Genomics, CA, USA), as previously described, and datasets used in our previous analyses from prior publications were reanalyzed in the context of the current study [9,39].

#### 4.1.6. Analysis of Spatial Sequencing Data

The 10× Genomics Visium platform was used for spatial transcriptomic profiling following previously established protocols [9]. Reads were demultiplexed and aligned to the hg38 reference genome using 10× Space Ranger v.1.3.1 (10× Genomics, CA, USA), employing default parameters for automatic alignment. Spatial data were loaded into count matrices using Seurat v4.0, retaining spots with less than 10% of transcripts mapping to mitochondrial genes for scaling and normalization of gene expression measurements via the sctransform method. Selected genes were utilized to annotate immune cells, including *PECAM1* and *ERG* for tumor cells; *FBLN1*, *FAP*, and *DES* for fibroblasts; *CD79A* and *CD79B* for B-cells; *CD8A* and *CD8B* for CD8 T cells; *KLRK1* and *KLRD1* for NK cells; *CD14* and *CD68* for macrophages; *CSF3R* and *FPR1* for neutrophils; *CD209* and *CCL13* for dendritic cells; and *TPSAB1*, *MS4A2*, *CPA3*, and *HDC* for mast cells.

#### 4.1.7. Statistics

*SPP1* expression levels were dichotomized into high and low categories using the median split method. The expression of *SPP1* in primary tumor samples was compared with various clinicopathological data, including epithelioid histology, Fédération Nationale des Centres de Lutte Contre le Cancer (FNCLCC) grading, tumor mutational burden (TMB), tumor inflammatory signature (TIS) scores, and survival outcomes. Progression-free survival was determined to be the period elapsed from diagnosis to either disease progression or death from any cause. Overall survival was calculated from diagnosis to death or censored at the last follow-up date for surviving patients. Using Kaplan–Meier analysis and Cox proportional hazards models, survival analyses were conducted with censoring applied at the last follow-up date. Box-and-whisker plots were used to represent continuous data, and associations with categorical variables were analyzed using the Mann–Whitney U or Kruskal–Wallis tests, as applicable. All statistical analyses were performed using MedCalc (Windows version 19.0.4), with the statistical significance threshold set at two-tailed *p* < 0.05.

#### 4.1.8. Study Approval

Written informed consent for the use of biospecimens and clinical data was obtained in compliance with the Declaration of Helsinki. Approval for this work was granted by the SingHealth Centralized Institution Review Board (CIRB2018/3182). All methods were carried out per the appropriate guidelines and regulations.

## Figures and Tables

**Table 1:** Angiosarcoma patient characteristics stratified by high and low *SPP1* levels.

		*SPP1* Levels			Total
		High	Low	*p*	
n (%)					72 (100%)
Sex					
	Male	21 (58.3%)	26 (72.2%)	0.219	47 (65.3%)
	Female	15 (41.7%)	10 (27.8%)		25 (34.7%)
Age at diagnosis (years)					
	<65	21 (58.3%)	15 (41.7%)	0.16	36 (50.0%)
	≥65	15 (41.7%)	21 (58.3%)		36 (50.0%)
Ethnicity					
	Chinese	27 (75.0%)	31 (86.1%)	0.237	58 (80.6%)
	Other	9 (25.0%)	5 (13.9%)		14 (19.4%)
FNCLCC tumor grade					
	3	13 (36.1%)	6 (16.7%)	0.005	19 (26.4%)
	2	19 (52.8%)	14 (38.9%)		33 (45.8%)
	1	4 (11.1%)	16 (44.4%)		20 (27.8%)
Site of primary tumor					
	Head and neck	17 (47.2%)	25 (69.4%)	0.058	42 (58.3%)
	Others ^a^	19 (52.8%)	11 (30.6%)		30 (41.7%)
Human herpesvirus-7					
	Positive	25 (69.4%)	22 (61.1%)	0.461	25 (34.7%)
	Negative	11 (30.6%)	14 (38.9%)		47 (65.3%)
UV signature					
	Present	11 (30.6%)	10 (27.8%)	0.797	51 (70.8%)
	Absent	25 (69.4%)	26 (72.2%)		21 (29.2%)
Epithelioid histology					
	Present	23 (63.9%)	13 (36.1%)	0.019	36 (50.0%)
	Absent	13 (36.1%)	23 (63.9%)		36 (50.0%)
Disease state at diagnosis ^b^					
	Metastatic	14 (38.9%)	8 (22.9%)	0.147	22 (31.0%)
	Non-metastatic	22 (61.1%)	27 (77.1%)		49 (69.0%)

^a^ Others: breast (n = 7), liver (n = 4), abdominal wall (n = 2), arm (n = 2), forearm (n = 2), peritoneum (n = 2), small bowel (n = 2), brachial plexus (n = 1), chest wall (n = 1), lower limb (n = 1), pleura (n = 1), prostate (n = 1), retroperitoneum (n = 1), spleen (n = 1), thigh muscle (n = 1), and vagina (n = 1). ^b^ Unknown disease state: n = 1.

## Data Availability

The NanoString gene expression profiling data used in the current study are available in GEO under accession no. GSE226338 and GSE227469.

## Supplementary Materials

The following supporting information can be downloaded at: https://www.mdpi.com/article/10.3390/ijms251910863/s1.
